# Electrophysiological and hemodynamic mismatch responses in rats listening to human speech syllables

**DOI:** 10.1371/journal.pone.0173801

**Published:** 2017-03-14

**Authors:** Mahdi Mahmoudzadeh, Ghislaine Dehaene-Lambertz, Fabrice Wallois

**Affiliations:** 1 INSERM U1105, GRAMFC, Université de Picardie Jules Verne, CHU SUD Amiens, Amiens, France; 2 Cognitive Neuroimaging Unit, CEA DSV/I2BM, INSERM, CNRS, Université Paris-Sud, Université Paris-Saclay, NeuroSpin center, Gif/Yvette, France; University of Jyväskylä, FINLAND

## Abstract

Speech is a complex auditory stimulus which is processed according to several time-scales. Whereas consonant discrimination is required to resolve rapid acoustic events, voice perception relies on slower cues. Humans, right from preterm ages, are particularly efficient to encode temporal cues. To compare the capacities of preterms to those observed in other mammals, we tested anesthetized adult rats by using exactly the same paradigm as that used in preterm neonates. We simultaneously recorded neural (using ECoG) and hemodynamic responses (using fNIRS) to series of human speech syllables and investigated the brain response to a change of consonant (ba vs. ga) and to a change of voice (male vs. female). Both methods revealed concordant results, although ECoG measures were more sensitive than fNIRS. Responses to syllables were bilateral, but with marked right-hemispheric lateralization. Responses to voice changes were observed with both methods, while only ECoG was sensitive to consonant changes. These results suggest that rats more effectively processed the speech envelope than fine temporal cues in contrast with human preterm neonates, in whom the opposite effects were observed. Cross-species comparisons constitute a very valuable tool to define the singularities of the human brain and species-specific bias that may help human infants to learn their native language.

## Introduction

Cross-species comparisons are crucial to understand the brain specificities of each species. Humans are particularly efficient in terms of oral communication, but the neural architecture underlying this behavior has not been fully elucidated. Humans identify two main types of information during verbal exchanges. They use voice particularities to recognize other members of the group, in the same way as many other species, but they also produce and understand complex and novel messages by means of a productive combinatorial system of elementary bricks, the phonemes. Phonetic and voice representations are progressively elaborated along parallel streams in the superior temporal region beyond the primary auditory cortex. Phonetic analyses are performed in the posterior superior temporal region biased toward the left hemisphere, whereas voice identification is performed more efficiently by the right hemisphere, in a more anterior region of the superior temporal lobe [1–3]. Because correct phonetic encoding requires shorter temporal windows for processing than voice identification, these hemispheric differences have been explained by differences in the sensitivity of each hemisphere to temporal modulations [4–6].

Human infants present similar hemispheric biases for phonetic perception and voice identification to those observed in adults [7, 8]. Furthermore, three months before term, at an age when neurons are still migrating to reach their final position in the cortical plate, preterm neonates already robustly perceive the difference between /ba/ and /ga/, whereas they only weakly react to a change of voice (male vs female voice) [9, 10]. The phonetic difference between /b/ and /g/ depends on a brief time-interval (45 ms in these stimuli) and therefore requires fine temporal resolution to process the frequency pattern. By contrast, the male/female voice difference is mainly based on the voice pitch carried by the entire syllable (285 ms in the stimuli). These results suggest that humans might benefit from a genetically driven ability for fine temporal coding of the auditory world, tailored to the acoustic structure of speech. However, if the same results are also observed in another species, they would support a more general hypothesis based on the acoustic features of the syllables combined with generic auditory processing capacities.

We therefore used the same paradigm in urethane-anesthetized rats as that used in human preterm infants [9, 10]. Adult rats and preterm infants are obviously different, but the purpose of this study was to try to reproduce an advantage for rapid temporal processing, which is crucial to perceive the difference between /b/ and /g/, over voice processing, possibly associated with hemispheric processing biases in a species other than humans to test the hypothesis of species-specific vs general auditory encoding processes. Numerous behavioral experiments comparing speech perception capacities in infants and animals have revealed surprising similarities. For example, just like human neonates, rats [11] and tamarins [12] discriminated sentences belonging to two different human languages. These species can also extract words from a speech stream using statistical computations [13, 14]. Chinchillas categorically perceived a voice-voiceless phonetic contrast with a similar “phonetic” boundary [15] to that of humans. Fewer studies have examined the neural bases of these behaviors and their similarities with human responses. Such studies have mainly explored sound discrimination and used oddball paradigms, in which a rare auditory stimulus is presented in a series of repeated sounds. When the change can be detected, the novel sound elicits a mismatch response, called mismatch negativity (MMN) in human adults. This is an early response (around 100–200 ms), the amplitude and scalp topography of which depend on the distance between the novel sound and the repeated sound [16, 17]. This response is recorded in sleeping [18, 19], comatose and anesthetized human subjects [20], suggesting automatic encoding of the sound features. An equivalent of the human MMN has been reported in many species: rats [21–27], mice [28], rabbits [29], guinea-pigs [30, 31], pigeons [32], dogs [33] and monkeys [34], and also in anesthetized animals [21, 24, 35–39]. This response therefore constitutes a valuable model to study similarities and differences in sound encoding between species.

The secondary objective was to compare electroencephalography and functional near-infrared spectroscopy (fNIRS), and their respective sensitivity in a cognitive task in rats. EEG directly measures neural responses, whereas fNIRS is dependent on neurovascular coupling, but fNIRS more accurately localizes active regions, situated underneath the optodes detecting the effect, than scalp EEG because of the mixing of the electrical fields that diffuse away from the source. We therefore constructed a recording system which simultaneously records both responses by means of electroptodes containing an electrode and a light emitter/detector. Oxy- (HbO) and deoxyhemoglobin (Hb) absorb light at different specific wavelengths. It is therefore possible to measure changes in HbO and Hb concentrations in the vessels surrounding an active cortical area and to infer the neural response to external stimuli in the cortex coarsely located between a light emitter and a detector [40–42]. As the electroptodes were directly placed on the rat brain, both techniques should give precise locations, allowing comparison of the sensitivity of the two methods by avoiding the distortion due to the skull.

Both techniques were previously used in preterms [9, 10] using an oddball design taking into account the constraints of these two techniques: brief stimuli for ERPs, but long stimulation periods to elicit a robust hemodynamic response for fNIRS. Syllables were therefore presented during blocks of 20 s followed by 40s of silence to allow the vascular response to develop and return to baseline. The hemodynamic response during standard blocks in which the syllable was always the same was compared to blocks in which either the voice producing the syllable was changed (deviant voice blocks) or the phoneme was changed (phoneme deviant blocks). Within each block, the syllables were presented by series of four. The first three syllables of the series were always the same during a given block (e.g. female /ba/ i.e. /ba_f_/), but the last syllable was either the same (standard trials: e.g. /ba_f_//ba_f_//ba_f_//ba_f_/) or different (deviant trials). In deviant voice blocks, the change concerned the voice (e.g. /ba_f_//ba_f_//ba_f_//ba_m_/) and in deviant phoneme blocks, the change concerned the phoneme (e.g. /ba_f_//ba_f_//ba_f_//ga_f_/). This design allowed us to study the response to repetition, which is expected to produce a decrease in neural activity (repetition suppression), whereas the novel syllable, if the difference is perceived, is expected to induce recovery of neural activity in regions coding the feature that has changed [5, 6]. This pattern has been observed using ERPs [43], but also in local field potentials (LFPs,[44]), and single neuron spiking activity [45]. We therefore expected a difference between standard blocks and deviant blocks in fNIRS and between standard trials and deviant trials in ECoG recordings under the precise channels corresponding to the active regions coding voices and phonemes. It should be noted that, as each syllable was either standard or deviant depending on the block, comparisons between standard and deviant trials/blocks would therefore not be related to the syllable features but to the discrimination process itself.

Finally, to check our recording apparatus before running the speech task in rats, a control task was performed by using somatosensory stimulation. Responses to forepaw median nerves are well described: the median nerve projects to discrete areas of the contralateral somatosensory cortex with tight spatial neurovascular coupling between this brain area and its vascular compartments [46]. It should be mentioned that in the present study we do not report any new human data.

### Experimental procedures

#### Animals

Fourteen adult male Sprague–Dawley rats (mean body weight: 350–450 g) were housed under controlled conditions in the animal house of the Medical Faculty of the Jules Verne University of Picardy (Amiens, France). The rats were kept in a 12-h dark/light cycle environment at a temperature of 22°C and had *ad libitum* access to standard chow and tap water. Rats were prepared according to experimental procedures complying with the Guidelines for Small Animal Research approved by the University of Picardie Jules Verne Committee on the Ethics of Animal Experiments (Amiens, France). Every effort was taken to reduce the number of animals tested and to minimize their suffering. At the end of the experiment, each rat was overdosed with urethane and returned to the animal facility (platform agreement: B80-021-009), in compliance with procedures for sacrificed animals. The University of Picardie Jules Verne Committee on the Ethics of Animal Experiments approved the entire study (No. 121212–23).

#### Animal surgery

**Fig 1:**
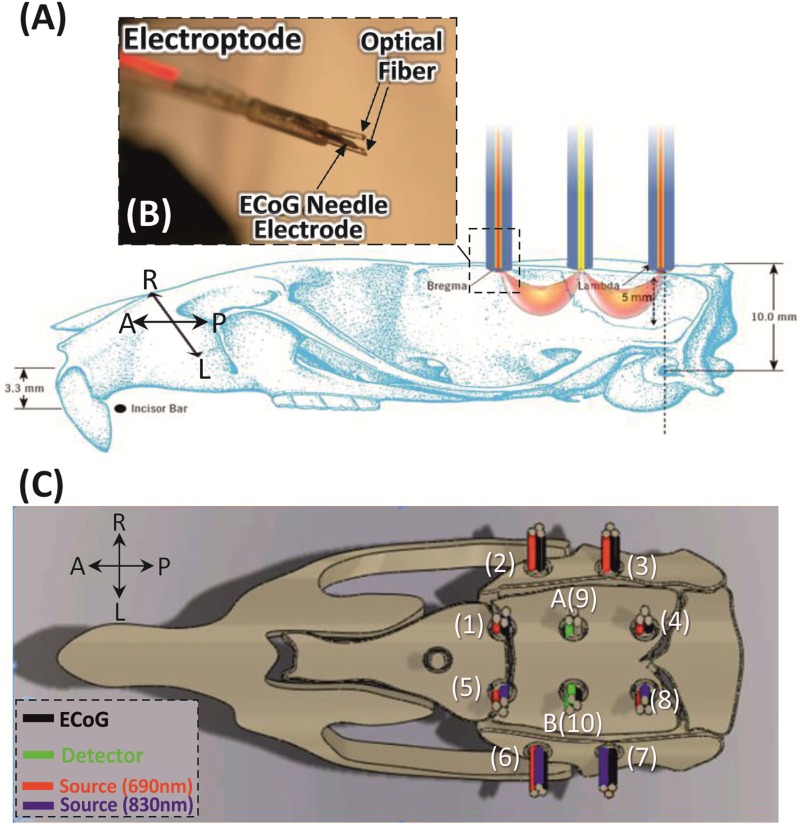
Experimental procedures. **(A)** Electroptode placement on a rat skull after drilling the scalp. Schematic drawing showing the position of the rat head during the recording with the crescent-shaped photon propagation of incoming light. **(B)** The custom-made electroptode consists of two optical fibers (400 μm core diameter, two wavelengths: 690 and 830 nm) and one ECoG electrode. **(C)** Top view of the electroptode configuration. The 10 electrodes coupled with 8 emitters and 2 detectors (A and B) resulting in 10 spectroscopy channels were arranged in a circle with a diameter of about 5 mm over the rat’s left and right hemispheres.

Prior to surgery, animals were anesthetized with urethane (1.25 g/kg, intraperitoneally) with additional doses (0.2 mL) administered during the test if necessary. The temperature was maintained at 37°C with a heating blanket pad. The rats were placed in a stereotactic frame. After a horizontal scalp incision, a 20×15-mm field of bone was exposed. The two lateral temporalis muscles were removed and eleven craniotomy holes (about 1.8 mm in diameter) were drilled relative to fiducial biomarkers (bregma and lambda, Fig 1) covering the auditory and somatosensory areas. The rats were then completely isolated from all external light sources by an opaque chamber to avoid interference of ambient light on the optical imaging recordings.

#### Electroptode configuration

To obtain simultaneous electrophysiological and optical measurements, we designed a special electroptode to hold the light emitter/detector fibers and ECoG electrodes over the small rat head. The standard electroptode can hold two optical fibers (core diameter: 400 μm) and one needle electrode (stainless steel, COMEPA^®^).

We used two detectors and eight light emitters and ten ECoG electrodes. These elements were arranged in a round grid pattern with 5 mm separation between nearest neighbor emitter–detector pair in order to obtain measurements from the 10 channels simultaneously, covering an area of about 100 mm^2^ = 10 mm (length) x 10 mm (width) in each hemisphere. The electroptodes entered the holes orthogonally to the tissue examined (Fig 1) and were secured to the rat’s skull via the custom-made minichamber. As each electroptode was positioned in a particular hole, their relative positions did not change from subject to subject and were accurately recovered for data analysis. The electroptode cables (optical fibers and electrode wires) were run through the round grid base window of the minichamber from where they were guided to the rat skull. Two 2.2 m long glass fibers (inner diameter: 3 mm) collected the scattered light and carried it to the PMT detectors. Each emitter consisted of two optical fibers (Fig 1B), one for each wavelength (690 and 830 nm).

### Recordings

#### ECoG

ECoG was amplified by a DC amplifier A.N.T^®^ (Enschede, The Netherlands) and recorded with a *frontal* reference at a sampling rate of 2048 Hz. The electrode impedance was kept below 1 kΩ.

#### Optical imaging

A multichannel frequency-domain-based optical imaging system (Imagent frequency-domain tissue spectrometer, ISS Inc., Champaign, IL) was used to acquire oxygenated hemoglobin and deoxygenated hemoglobin concentration changes. We used 8 of the 32 intensity-modulated laser diodes at two wavelengths (λ = 690 nm and 830 nm) coupled to optical fibers, and 2 of the 4 gain-modulated photomultiplier tube (PMT) detectors to separately collect the signal at the two wavelengths. The modulation frequency of laser intensity was 110 MHz, and the cross-correlation frequency for heterodyne detection was 5 kHz. Reflected light was collected in photomultiplier tubes (PMT) and demodulated. The mean intensity (DC), modulation amplitude (AC), and phase of reflected light were determined. The average output power of the lasers was about 0.5 mW and the optical system acquisition rate was 9.1912 Hz (about one sample every 110 ms). Oxy (HbO) and deoxyhemoglobin (Hb) are chromophores that absorb light at different wavelengths. The Modified Beer Lambert Law was applied to the two wavelengths (690 and 830 nm) to convert signal intensities into relative changes in (de)oxy-hemoglobin concentration.

### Stimulation protocols

#### Somatosensory stimulation

**Fig 2:**
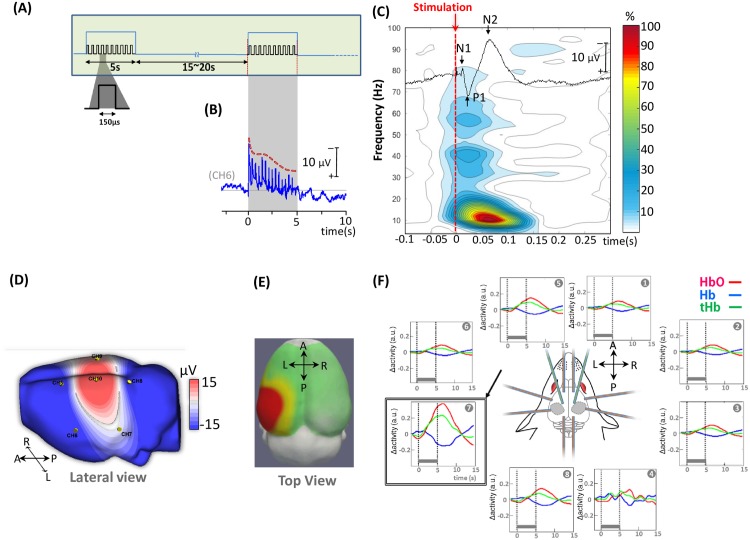
Neuronal and hemodynamic responses during the somatosensory task. **(A)** Somatosensory stimulation paradigm. Electrical stimulation was delivered for 5 s at 3 Hz (150 μs square-wave pulse) with a pseudorandom 15–20 s inter-trial interval via stainless steel electrodes. **(B)** Grand average of somatosensory cortical response over 5 s of electrical stimulation (CH6); a decreased amplitude was observed with repetition of forepaw nerve stimulation. **(C)** Typical ECoG neuronal response to electrical stimulation of the right forepaw and its time-frequency representation. **(D)** ECoG signal of the cortical response to electrical stimulation of the right forepaw in an individual rat and topographical mapping of the P1 component on a rat head model (extracted from rat MRI, http://expmr.ki.se/research/ratatlas.jsp) of the response. **(E)** Topography of HbO activation and **(F)** Cortical hemodynamic responses after grand averaging over 14 rats (total hemoglobin (tHb, green), oxyhemoglobin (HbO, red) and deoxyhemoglobin (Hb, blue)).

Electrical stimulation was delivered by using an electrical stimulator (IRES-600, Micromed^®^, Italy) at the motor threshold to the right rat forepaw via stainless steel electrodes for 5 s at 3 Hz (150 μs square-wave pulse) with a pseudorandom 15-20s inter-trial interval. Thirty trials, representing 450 stimuli, were used (Fig 2A).

#### Auditory stimulation

The stimulation procedure used in the preterm study was also used in this study [9]. Four digitized syllables, naturally produced by a French male (ba^**m**^, ga^**m**^) and a French female speaker (ba^**f**^, ga^**f**^), were matched for intonation, intensity, total duration (285 ms), prevoicing and voiced formant transition duration (40 and 45 ms, respectively).

**Fig 3:**
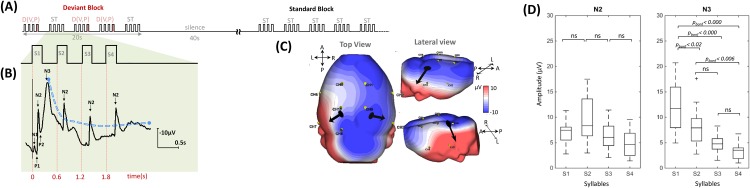
Auditory stimulation paradigm. **(A)** The syllables were presented by series of four (SOA = 600 ms) to form three types of trials (standard, deviant voice and deviant phoneme trials). Three types of block were constituted and presented randomly separated by 40 s of silence for a total duration of 108 min. **(B)** Typical one-channel auditory cortical response illustrates the decrease of averaged evoked response from the first to the fourth stimuli in one rat. The first syllable elicited 5 peaks (N1, P1, N2, P2, and N3) occurring at 3± 3 ms, 74± 3 ms, 135±3 ms, 195± 8 ms and 350± 10 ms, respectively. Underlying the fast components, a slow-wave was observed. **(C)** N2 topography in a typical rat. **(D)** Mean amplitude across all rats of the N2 and N3 peaks derived from the ERP waveforms of all channels, which were analyzed by one-way ANOVA with *post hoc* Bonferroni multiple comparison testing.

The syllable presentation was designed to fit both ERP and BOLD response constraints. Syllables were presented by series of four (SOA = 600 ms) to form three types of trials (standard, deviant voice and deviant phoneme trials). In standard trials, the same syllable was repeated four times (e.g. ga^**m**^ ga^**m**^ ga^**m**^ ga^**m**^). In deviant trials, the last syllable changed relative to the first three syllables either along the voice dimension (e.g. ga^**m**^ ga^**m**^ ga^**m**^ ga^**f**^), or along the phonetic dimension (e.g. ga^**m**^ ga^**m**^ ga^**m**^ ba^**m**^). Trials were regrouped in blocks of 20 s (5 trials separated by an inter-trial interval of 1600 ms, Fig 3A) followed by 40 s of silence for a total duration of 108 min. Three types of blocks were constituted and presented randomly. In standard blocks (ST), all trials were standard trials. Deviant blocks always began with a deviant trial, then two standard and two deviant trials were randomly intermixed. Deviant trials were along the voice dimension in DV blocks, and along the phonetic dimension in DP blocks. In each block, the repeated syllable was kept constant and was randomly chosen from among the four possible syllables (ba^**m**^, ga^**m**^, ba^**f**^ and ga^**f**^) in order to present each syllable the same number of times in each condition and to each rat. Stimuli were presented binaurally at a standard hearing level (≈ 70 dB) via speakers placed at a distance of 30 cm from the rat’s head.

The block design allowed analysis of the vascular response in the presence or absence of a change (standard blocks *vs*. each type of deviant block). Although only 3 of the 20 syllables of the deviant blocks were different, these differences were sufficient to observe an increase in HBO in preterm infants [9], but also an increase in the fMRI BOLD signal in adults [47] in a set of perisylvian areas. Presentation of syllables by series of 4 allowed analysis of the neural response to repetition (i.e. ERPs to the first three syllables) and to a change by comparing the response to the same last syllable depending on the preceding syllables in the various types of trials. Infants [43] and adults [48] display a voltage decrease with repetition and a hierarchy of mismatch responses following a change: an early response, mainly originating in secondary auditory areas [49], is followed by a later response (P300 in adults, late slow wave in infants). The scalp topography of the early mismatch response depends on the auditory/phonetic feature which is changed, demonstrating that different sets of areas are involved in the coding of different sound features in humans. In particular, detection of a change of articulation, such as /ba/ to /ga/, depends on accurate temporal coding, mainly involving left hemispheric regions, whereas a male to female voice change depends on spectral representations mainly in the right hemisphere.

### Data processing and auditory task analysis

#### ECoG

ECoG signals were down-sampled to 1024 Hz and filtered with a 0.05–20 Hz band-pass filter (high pass: 12 dB/octave, zero-phase; low pass: 24 dB/octave, zero-phase). As recording the DC cortical field potential provides a more accurate picture of the actual functional status of neuronal cells [50], analyses were performed using DC data (>0.05 Hz). Data were segmented and baseline corrected, starting 100 ms before the onset of stimulation and ending 300 ms after the onset of somatosensory stimulation and 4000 ms after the onset of auditory stimulation. Artifact rejection was based on visual inspection. Trials with high amplitude (>200μV), linear trends, and with artifacts related to the electronics such as electrode ‘jumps’ or ‘spikes’ were visually rejected. The original number of trials was 144/108/108 for ST/DV/DP trials and 118/82/84 after artifact rejection. Artifact-free epochs were averaged in each rat according to conditions and a grand average was computed across all rats in each condition.

**GMFP:** Global Mean Field Power was used to characterize the neural response to repetition and novelty, which may diffuse over several electrodes (GMFP, [51]). GMFP is the standard deviation of the potentials at all electrodes of an average reference map calculated at each time point using the following formula:

$$\text{GMFP}\left(\text{t}\right)=\sqrt{\frac{\sum_{\text{i}}^{\text{k}}{\left({\text{V}}_{\text{i}}\left(\text{t}\right)-{\text{V}}_{\text{mean}}\left(\text{t}\right)\right)}^{2}}{\text{K}}}$$ (1)

where t is time, V_i_ is the voltage at channel i, V_mean_ is the mean of the voltages in all channels and K is the number of channels. This parameter is commonly used to identify time-samples with a high signal-to-noise ratio, which is related to periods of increased global neuronal synchronization [52, 53]. The larger the GMFP, the more intense is the response. The advantage of GMFP is that it takes all electrodes into account without any *a priori* assumptions on the location of the response and on the diffusion of the electric field and therefore provides an unbiased measure of neural activity [54].

We first identified the peaks following the first syllable of the trial as the maxima of the GMFP computed on the grand-average for all rats. Second, to study the effect of syllable repetition, we determined the maximum GMFP in each rat in time windows centered on these peaks (N2: 100–150 ms and N3: 300–600 ms) after each syllable and entered these values into separate analyses of variance (ANOVAs), using syllables (S1, S2, S3 and S4) as within-rat variables. We report *post hoc* t-tests with Bonferroni correction. Third, to study the response to a change, we entered all GMFP values between S4 onset until 1200 ms post-S4, in two ANOVAs in which each deviant condition was compared to the standard. A significance level (p_(uncorr)_< 0.025) is reported for each comparison (DV *vs*. ST, DP *vs*. ST).

**Time-frequency analyses:** We then analyzed the time-frequency decomposition of each ECoG trial [55] using BESA^®^ software (BESA GmbH, Gräfelfing, Germany;[56]). Analyses of evoked responses only detect electrical activity that is precisely time-locked to the stimuli, whereas time-frequency analyses may detect more variable responses, such as bursts induced by the stimulus. Time-frequency analyses indicate which frequencies present the highest powers at specific points in time and space and how their phase angles synchronize across time and space. Because ECoG rhythms are themselves the product of synchronized activity among and within neuronal assemblies, it is often assumed that changes in ECoG power reflect underlying changes in neuronal synchrony. Time-frequency displays, which represent the change in amplitude over time (TFR, Time-Frequency Representation), were generated from the single trials by averaging spectral density amplitudes over trials and normalizing to the baseline for each frequency and each channel.

$$\text{TFR}=\frac{\text{A}\left(\text{t},\text{f}\right)-{\text{A}}_{\text{baseline}}\left(\text{f}\right)}{{\text{A}}_{\text{baseline}}\left(\text{f}\right)}.100\text{\%},$$ (2)

where *A*(*t*, *f*) = activity at time *t* and frequency *f* (absolute amplitude) and *A*_*baseline*_(*f*) = mean activity at frequency *f* over the baseline epoch. TFR were limited between 2 and 20 Hz (step of 2 Hz) and down-sampled to a time sample every 25 ms for display and statistical analyses.

The TFR cluster-randomization approach implemented in BESA Statistics 1.0 (BESA GmbH) was used to compare the time-frequency decomposition in each condition and correct for type I errors potentially arising from multiple comparisons. This data-driven procedure is particularly useful for spatial localization without having to specify regions of interest *a priori* [57–61]. After having computed the t-test between two conditions at each time-point, frequency and channel, statistic clusters were constituted as the sum of the t-values above a given threshold (in this case p≤0.05) in neighboring time-points, channels and frequencies. To test the probability of significance of these clusters, the null distribution was computed from the data by randomly assigning the condition label to either of the two conditions and performing the same computation of statistic clusters for each of 1000 permutations. Finally, the cluster p-value was determined by ranking the observed cluster relative to the distribution of surrogate clusters determined with the permutations.

#### Optical data

Optical data were band-pass filtered [0.03–0.5 Hz] using a zero-phase filter (Butterworth, order: 6) to eliminate physiological noise (e.g. slow drifts, arterial pulse oscillations). HbO and Hb signals were segmented relative to the onset of each block (-5 to +25s). After applying a linear detrend and baseline correction, the segments were averaged by condition (3 conditions) and channel (8 channels) in each rat (average number of blocks per condition and channel are 35/34/35 for ST/DV/DP).

Hemodynamic responses were analyzed on the Area Under the Curve (AUC) of averaged Hb, HbO signals. In order to test whether syllables elicited stronger lateralized responses in the rat auditory cortex, we subsequently performed between-hemispheric comparisons using Wilcoxon Holms-adjusted t-tests.

## Results

### Somatosensory cortical responses

A prominent somatosensory electrical potential over the contralateral rat cortex (Channel 10), with its classical three components (mean±SD; N1: 14± 3 ms, P1: 25± 5 ms and N2: 64± 7 ms, Fig 2C and 2D) was recorded in response to electrical stimulation of the right forepaw. Hemodynamic responses (segments: -5 to +15s) were also locally evoked in the somatosensory cortex with a maximal response recorded on channel 7 (Fig 2E). The mean hemodynamic response was monophasic, reaching a maximal change approximately 6±1s after stimulus onset and returning to baseline after an additional 5±2 s (Fig 2E). No evidence of an initial dip was visible. These results demonstrate satisfactory and simultaneous recording of both neural and hemodynamic signals with congruent topographies. Since fNIRS probes the inter-electroptode cortical area (e.g. light source on CH7, detector on CH10), the maximum neural response on electrode 10 with inversion of polarity on electrode 7 is congruent with the maximum fNIRS response located on the same channel 7.

### Auditory cortical responses: Response to syllable repetition

The first syllable elicited 5 peaks (N1, P1, N2, P2, and N3) occurring with a maximum amplitude on channel 3 (right auditory cortex) at 33± 3 ms, 74±3 ms, 135± 3 ms, 195±8 ms and 350± 10 ms, respectively (Fig 3B) on top of a slow wave, starting at the onset of the first syllable and returning to baseline about 1.2 s later (0.41 Hz). As shown in Fig 3D, N2 peaks remained clearly visible after each repetition of the syllable, whereas N3 amplitude decreased with subsequent repetitions (F_(3,52)_ = 16.93, p < 0.001). *Post hoc* Bonferroni pairwise comparisons showed that the N3 amplitude for S1 was significantly higher than for any other syllable (S2: p_(bonf.)s_<0.02; S3<0.000; and S4<0.000). In contrast, no significant difference was observed for N2 after S1 and after S2, S3, S4 under the standard condition (Fig 3D).

### Mismatch response to auditory change

The strongest mismatch response in terms of GMFP was observed between 493 and 573 ms for voice change and between 360 to 650 ms for consonant change (*ps*_*(uncorr)*_ *< 0*.*05*). These latencies corresponded to the N3 component.

**Fig 4:**
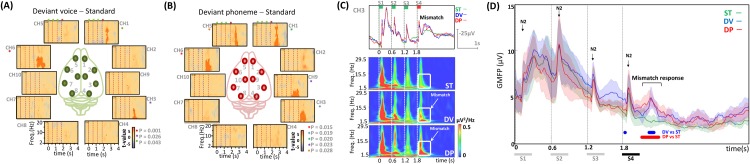
Auditory mismatch response. **(A-B)** Comparison of the time-frequency decomposition for each deviant vs. standard trial. Pale red colors represent positive clusters (i.e. deviants had larger t-values than standard conditions) and negative clusters are marked with light blue colors (i.e. deviants had smaller t-values than standard conditions). Significant positive clusters are marked with pale red color. Each cortical area is dominated by activity in the theta to beta range over a certain period of time. The probability level under which the specific cluster is significant is shown in the right bottom corner. Red dotted vertical line indicates the onset of each of the four syllables. **(C)** Time-course of the grand average ECoG signal of three conditions (ST, DV, DP) showing habituation in terms of amplitude. The mismatch response is observed in time-domain and ECoG time-frequency maps of three different conditions. **(D)** Time-courses of the grand average and standard deviation of Global mean field power (GMFP) in deviant phoneme (DP), deviant voice (DV), and standard (ST) trials. The peaks corresponding to N2 are indicated. The strongest mismatch response was observed over the period 300 to 800 ms, corresponding to the N3-evoked potential component (horizontal lines are plotted when significant differences are observed; DV vs. ST blue color, DP vs. ST red color). The gray rectangles indicate the duration of the syllable.

The permutation test on the Time-Frequency Representations (TFRs) of deviant and standard conditions revealed a significant difference over the right auditory area (Fig 4A and 4B; CH3) and over the right and left frontal areas (CH1 and CH6) from 318 to 1200 ms for both DV and DP compared to the standard condition (for DV: CH1: p_clusterDV_ = 0.043, CH3: p_clusterDV_ = 0.026, CH6: p_clusterDV_ = 0.001 and for DP: CH1: p_clusterDP_ = 0.02; CH3: p_clusterDP_ = 0.019, CH6: p_clusterDP_ = 0.015). In addition, two other significant clusters that appeared to be dominated by activity in the delta to beta range were observed for DP (CH9: p_clusterDP_ = 0.023 and CH5 p_clusterDP_ = 0.028) compared to a less extensive area in DV vs the standard condition. Nevertheless, no significant TFR difference was observed when comparing the two deviant conditions. In both cases, an increase in delta-beta frequency amplitude relative to the standard condition was observed with a maximum around 300–800 ms (see Fig 4C for an example in a typical rat on channel 3).

### Local hemodynamic response to auditory stimuli

**Fig 5:**
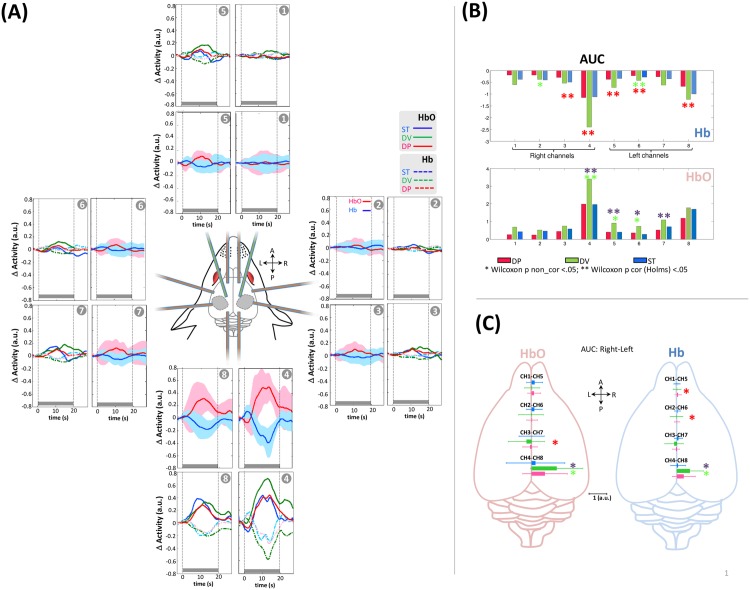
Auditory hemodynamic responses. **(A)** Mean auditory hemodynamic response in each channel after pooling the three different conditions (deoxyHb in blue, oxyHb in red) and when they were separated (HbO). The gray rectangle indicates the duration of the stimulation block. **(B, C)** Area under the curve of hemodynamic response in the various channels and conditions. B) Comparisons between conditions are performed two by two in each channel.*P < 0.05, **Pcorr < 0.05. Channels 1–4 are situated over the right hemisphere (RH) and channels 5–8 are situated over the left hemisphere (LH). (* significant difference between DV vs. ST, * significant difference between DP vs. ST, * significant difference between DP vs. DV) **(C)** Comparison of between-hemisphere AUCs of hemodynamic responses at homotopic locations.

The grand average of the hemodynamic response related to the block stimuli induced typical neurovascular coupling in all channels (Fig 5A). The hemodynamic response was maximal over channels 4 and 8 with a significant rightward asymmetry for both Hb and HbO (Fig 5C, CH4 > CH8 p_corr(Holms)_ < 0.05) and a maximum peak latency around 10±2 sec (Fig 5A). Furthermore, in CH4 and in all conditions, responses were biphasic and started with an initial dip characterized by a short (0–4 sec) negative deflection of HbO and a positive deflection of Hb, followed by bilateral overflow associated with an increase in HbO and a simultaneous decrease in Hb.

No significant difference was observed between DP and standard blocks, whereas a change of voice induced a significant increase in HbO and Hb on the right channel CH4 (Fig 5C, CH4 and CH8 over the auditory cortices, p_corr (Holms)_ <0.05), resulting in a significant difference between DP and DV blocks on the same channel.

### Relationship between the initial hemodynamic dip and the DC component in ECoG

**Fig 6:**
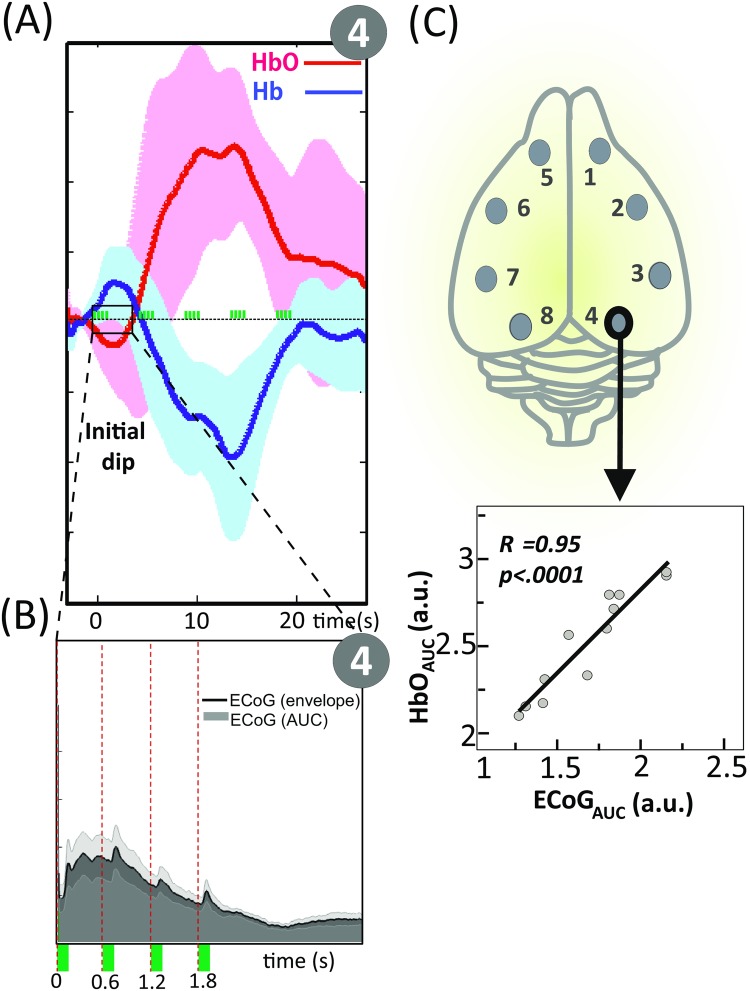
Relationship between the initial hemodynamic dip and the slow shift of ECoG. **(A)** Time-course of the grand average Hb (blue line) and HbO (red line) across all conditions (Ch. 4). The y axis represents the concentration changes in arbitrary units (a.u.). The green rectangles indicate stimulation. **(B)** Time-course of the ECoG grand average signal and its amplitude envelope (Hilbert transformed). **(C)** Scatter plot of the area under the curve of the initial HbO dip [0 to 4 sec] plotted against the ECoG amplitude envelope (Hilbert transformed). The correlation coefficient and related P value are shown as an inset.

To study the relationship between the initial hemodynamic response and the slow shift of ECoG signal, the area under the curve of the initial HbO dip [0 to 4 sec, across all conditions] and the ECoG amplitude envelope (Hilbert transformed) were computed. We then calculated correlation coefficients across all eight channels for the combined hemodynamic and electrical datasets. A highly significant correlation [r = .95 (p <.0001)] was observed for channel 4 (Fig 6).

## Discussion

We simultaneously recorded ECoG-fNIRS signals in urethane-anesthetized rats in a syllable discrimination task to compare the responses of rats and preterm neonates [9, 10] and to evaluate the sensitivity of these methods to visualize cognitive processes. We first demonstrated that congruent neural and hemodynamic responses to rat median nerve stimulation can be recorded in the contralateral motor region, in total agreement with the results previously reported by [46]. These results validate our simultaneous EEG-optical imaging recording device. Similarly, we recorded auditory responses to speech syllables over auditory areas with both methods. The cortical auditory evoked response comprised 5 peaks, N1, P1, N2, P2, and N3, named according to their polarity over the auditory cortex. The latencies of these peaks are in agreement with previous reports [44, 62], although our stimuli were more complex and of longer duration than the tones used in these studies. An inversion of polarity for all peaks was observed on the top lateral part of the right and left hemispheres (Fig 3C lateral view), suggesting sources in the auditory cortices. These putative sources were confirmed by fNIRS recordings, which showed responses limited to the auditory areas. Both neural and hemodynamic responses were asymmetric in favor of the right hemisphere.

### Neural responses

After confirming the validity of our measurements, we examined how the rat brain reacts to repetition of the same syllable and to sudden changes. Our paradigm is a particular version of the classical oddball paradigms, in which stimulus repetition induces decreased neural activity (repetition suppression), whereas a sudden change causes recovery of neural activity in regions coding for the parameter that changed [43, 63]. In the present animal study, we observed that all components, except N2 (135 ms), showed a rapid decrease in amplitude, detectable from the first repetition, similar to that reported in human premature [64] and full-term neonates [18], 3-month-old post-term infants [43] and adults [49] using the same protocol. Various studies in the literature have questioned whether this decreased amplitude is related to stimulus adaptation and/or predictive coding. As the present paradigm is not designed to answer this question, this aspect will not be discussed in more detail.

The response to a change in the series was recorded at the latency of the N3 after 300 ms, which is much later than the MMN in human adults (about 100–250 ms), even after taking into account the fact that anesthesia may have slowed the rat’s responses. Several studies have reported similar late latencies of responses to an auditory change, even in awake rats, but inconsistent responses to auditory changes have been reported in the literature. These inconsistencies have been related to different choices of control stimulus (see discussion [25, 44, 65]), which is a difficult problem in classical oddball paradigms. In the classical auditory oddball paradigm, a sequence of repetitive standard sounds is interrupted infrequently by deviant “oddball” stimuli (eg. infrequent stimuli that differ in duration, frequency or pitch from the more frequently presented stimuli). Our experimental design avoids the disadvantages related to this paradigm: the deviant and standard syllables compared in this design were situated at the same last position in the trial. The inter-trial interval was sufficient to allow recovery of ERPs from adaptation and the context before the critical stimulus (number of stimuli and transitions) was similar. Furthermore, our study design allowed comparison of each syllable to itself at short time-intervals to avoid any time-related confounding, such as differences in the animal’s state. This type of paradigm, as opposed to the classical oddball, is therefore particularly efficient to eliminate numerous non-pertinent differences between control and deviant stimuli and to consequently reveal a genuine discrimination process.

Imada et al. [44] recorded LFP during a tone detection task in awake rats. The late deviance detection response, that they called P3L, was similar to the response observed in our study in terms of latency and topography, including a frontal component. The authors related this late response to the P300 recorded in attentive human adults. In the present study, an increase in delta-beta frequencies over frontal electrodes was clearly observed, but, because of the anesthesia, frontal activity was probably weak and did not affect the hemodynamic response (i.e. significant hemodynamic changes for deviant relative to standard blocks were only recorded over the auditory cortex). The rhythmicity of the stimulus presentation and the fixed position of the deviant stimulus at the last position of the trial may have facilitated a lock to the temporal structure of the stimulation and an automatic orientation to that precise moment [66, 67]. Automatic attention orientation to a novel stimulus may therefore still be elicited under anesthesia and the rat response recorded here might correspond to the automatically elicited human P3a, which is observed even when the subject’s overt attention is directed to another direction or another object.

### Neural vs. hemodynamic responses

The strength of our study is related to the simultaneous recording of neural and hemodynamic responses. Urethane, like other anesthetics, affects neurovascular coupling. Nevertheless, responses were generally congruent with both methods, but neural recordings were more sensitive, particularly to detect the response to a novel stimulus. Whereas significant differences with the standard condition were detected for a change of voice, only ECoG was sensitive to the change of phoneme, which is surprising in view of the similar strength and spread of the neural response for both types of changes (Fig 4). This discrepancy might be related to the fact that consonant encoding is more difficult than voice encoding in rats. The difference between /ba/ and /ga/ is related to the slope of the formant transition over a few tens of milliseconds, whereas different voices have different F0 and harmonics during the entire duration of the syllable. Furthermore, to study the consonant change, we merged blocks in which the voice was either male or female. Although phoneme discrimination in humans is equally easy for both male and female voices, a different situation may be observed in other species. Small pitch/envelope differences between productions that cannot be avoided with natural syllables may have been present, but the fact that these differences were not consistent across the male and female speakers could explain the weaker response to /ba/ /ga/ changes if rats do not use consistent slope differences in the same way as humans. Similarly rats that have learnt to discriminate sentences from two different human languages cannot generalize when novel voices are used, in contrast with human neonates, also suggesting species differences in the sensitivity to linguistic and voice features [68]. It should also be noted that, with our paradigm, a deviant block comprised only three out of five trials with a change on the last syllable (i.e. 3 deviant syllables out of 20 syllables). Although discrimination might have been variable for one trial to the next, it may nevertheless have been sufficient to be observed in the average ECoG across all trials, but may not have been sufficient to induce a neurovascular response during the block.

This result highlights interesting differences between anesthetized rats and human preterm neonates previously tested in the exact same paradigm. Unlike rats, human neonates displayed large and robust responses to a change of phoneme, whereas the responses to a change of voice measured by fNIRS and EEG were weak and poorly time-locked to the event onset, suggesting an immature response [9, 64]. In the previous human study the infants were tested ten weeks before term [9], at an age at which neurons are still migrating to their final location in the cortical plate and a transient circuitry between thalamic afferents and subplate neurons is in place. The different sensitivity of the two groups to these two types of changes demonstrate a particular sensitivity of the human brain, even at an immature stage, to the fast temporal features of speech sounds which may differ across species.

### Hemispheric asymmetries

A second difference between humans and rats concerns hemispheric asymmetries in response to speech syllables. The neural and hemodynamic responses to syllables were both right-lateralized in rats, in contrast with what is commonly observed in humans. Since the electrical and hemodynamic responses to right median nerve stimuli were well localized in the contralateral left somatosensory cortex and because the neuronal electrical responses to syllabic stimuli were recorded bilaterally (despite a right bias), it is unlikely that the lack of significant hemodynamic enhancement to syllabic stimuli on the left cortex in rats was due to technical problems (poor detector or emitter positioning). Hemispheric asymmetries favoring the right hemisphere, such as larger volume [69] and better spectral processing capacities [70], have been described in rats, especially in males. In contrast, in guinea-pigs [71], left-lateralized responses have been reported in the thalamus to the /da/ syllable, whereas the responses to tones and clicks were symmetrical. A left-hemispheric superiority has been reported when rats were required to discriminate whether two short (20 ms) tones presented in one ear were similar or presented different frequencies, while white noise was presented to the other ear [72] and when they had to discriminate a series of gaps in continuous noise under conditions of unilateral or bilateral reversible inactivation of the auditory cortex [73]. Hemispheric biases to the processing of auditory stimuli are therefore not specific to the human brain, but either the direction of asymmetry to process the same feature is species-specific or different species are not sensitive to the same acoustic features. When hearing human speech, rats might more effectively encode its slow acoustic features [74], such as the speech envelope and spectral slow modulation, rather than its faster temporal structure. This spontaneous bias may explain the stronger response for a voice change than for the consonant change, especially observed with optical recordings.

In preterm neonates [9], hemodynamic responses were faster and more prolonged in the left temporal region than in the right superior temporal region in the *planum temporale*. Activation in the left frontal region was modulated by a change of phoneme, whereas both changes affected the right frontal region. The observation that human infants present a left bias in the *planum temporale* [8] even before term [9] suggests that the human auditory system is prepared to process the fast features of speech, crucial to represent phonemes. As this phenomenon was not observed in rats, it does not constitute a general feature induced by the type of stimuli, but, on the contrary, this functional bias in processing auditory stimuli might be genetically determined, like several other structural asymmetries clearly observed in the human brain before term, now that brain images can be obtained at an early age (e.g. Yakovlevian torque, deepest right superior temporal sulcus [75]. We propose that the opposite hemispheric responses for exactly the same stimuli in exactly the same experimental paradigm observed in preterm neonates and rats could be related to a different bias toward the fast and slow components of speech. This observation needs to be confirmed in future studies. If validated, it might reveal a common functional division between the left and right hemispheres in mammals, with reinforcement of the left fast-temporal encoding processing stream in humans.

### Correlation between the initial dip and DC component

Before presenting our conclusions, we would like to describe a fortuitous but instructive observation on the relationship between neural and vascular responses. Although the major hemodynamic response to neural activation is a significant inflow of oxygenated hemoglobin to the activated region, a negative deflection in the HbO signal (‘initial dips’, peak < 2s following stimulation) was consistently detected in the auditory task in rat (Fig 6), but not during somatosensory stimulation. Although initially debated (see original review of [76]), initial dips have been related to an increase in oxygen consumption due to the oxidative metabolism of activated neurons starting 100 ms after the onset of electrical activity, which is subsequently compensated by the vascular response resulting in an overflow of HbO [77]. This pattern has been explained by two successive and overlapping mechanisms: first, increased oxygen consumption within a single column is followed by more widespread activation spreading over several columns [78]. Furthermore, in the case of optical imaging, due to the upstream propagation from the deepest to the more superficial layers of the vasodilation response and the higher sensitivity of optical imaging for the most superficial layers, the initial dip is more localized and more specific than the later hemodynamic response [79–82].

The presence of an initial dip has been reported over the whisker barrel cortex in some experiments [83], but not in others [84]. In the present animal study, the differences between auditory and somatosensory responses could be related to the complex auditory stimulation that may involve different cortical layers [81]. Not only were the auditory stimuli complex, but the paradigm based on predictions and signal errors should have notably involved supragranular layers, as proposed in different models of cortical responses notably during oddball paradigms [63, 85].

The initial dip was correlated with a negative DC shift in the neural recording above the same auditory region. DC shift is thought to represent sustained excitatory inputs to apical dendrites of cortical pyramidal cells and correlates with synaptic current loops and changes in glial cell potential [50, 86, 87]. In the present study, a DC shift occurred during the initial dip and was not associated with any change in blood volume, which at least partly contradicts the hypothesis according to which a contribution from the blood brain barrier-driven current generator is required to produce the millivolt-scale slow potential changes in EEG signal [88, 89]. The DC shift and the initial dip may both depend on local transient pH changes secondary to a rapid transient increase in the cerebral metabolic rate of oxygen (CMRO_2_), as we have previously observed in seizures [90]. Regardless of the exact relationship between these two phenomena, they indicate an activity in the superficial layers of the auditory cortex during the task, whereas the same phenomena were not observed during the somatosensory task. This relationship might be a signature of layer-specific activations that may also need to be considered in human studies.

## Conclusions

This animal study provides electrophysiological and hemodynamic data on the localization of rat cortical activity in a syllabic discrimination task. As in humans [9], the coding of syllabic stimuli is asymmetric, but in an opposite way (rightward hemispheric asymmetry in rats vs. leftward asymmetry in humans), suggesting that the two species do not spontaneously favor the same stimulus features. Inter-species comparisons in the same paradigms and with the same recording devices are crucial to understand human functional organization. The sensitivity to phonetic features and the left-hemispheric lateralization of some of the responses to speech in preterm neonates [9] are not observed in the rat, suggesting a specific sensitivity to auditory temporal features in the human species, which may facilitate speech learning.
